# Comparison of Portable and Bench-Top Spectrometers for Mid-Infrared Diffuse Reflectance Measurements of Soils

**DOI:** 10.3390/s18040993

**Published:** 2018-03-27

**Authors:** Christopher Hutengs, Bernard Ludwig, András Jung, Andreas Eisele, Michael Vohland

**Affiliations:** 1 Geoinformatics and Remote Sensing, Institute for Geography, Leipzig University, Johannisallee 19a, 04103 Leipzig, Germany; michael.vohland@uni-leipzig.de; 2 German Centre for Integrative Biodiversity Research (iDiv) Halle-Jena-Leipzig, Deutscher Platz 5e, 04103 Leipzig, Germany; 3 Deparatment of Environmental Chemistry, Kassel University, Nordbahnhofstr. 1a, 37213 Witzenhausen, Germany; bludwig@uni-kassel.de; 4 Technical Department, Szent István University, Villányi út 29-43, 1118 Budapest, Hungary; jung.andras@kertk.szie.hu; 5 SphereOptics GmbH, Gewerbestrasse 13, 82211 Herrsching, Germany; aeisele@sphereoptics.de

**Keywords:** portable FTIR spectrometer, mid-infrared soil spectroscopy, benchmarking, noise analysis, continuous wavelet transform, multivariate calibration, partial least squares, spectral variable selection

## Abstract

Mid-infrared (MIR) spectroscopy has received widespread interest as a method to complement traditional soil analysis. Recently available portable MIR spectrometers additionally offer potential for on-site applications, given sufficient spectral data quality. We therefore tested the performance of the Agilent 4300 Handheld FTIR (DRIFT spectra) in comparison to a Bruker Tensor 27 bench-top instrument in terms of (i) spectral quality and measurement noise quantified by wavelet analysis; (ii) accuracy of partial least squares (PLS) calibrations for soil organic carbon (SOC), total nitrogen (N), pH, clay and sand content with a repeated cross-validation analysis; and (iii) key spectral regions for these soil properties identified with a Monte Carlo spectral variable selection approach. Measurements and multivariate calibrations with the handheld device were as good as or slightly better than Bruker equipped with a DRIFT accessory, but not as accurate as with directional hemispherical reflectance (DHR) data collected with an integrating sphere. Variations in noise did not markedly affect the accuracy of multivariate PLS calibrations. Identified key spectral regions for PLS calibrations provided a good match between Agilent and Bruker DHR data, especially for SOC and N. Our findings suggest that portable FTIR instruments are a viable alternative for MIR measurements in the laboratory and offer great potential for on-site applications.

## 1. Introduction

In recent years, soil spectroscopy has been established as an efficient method to complement conventional soil analysis [1,2,3]. Compared to analytical laboratory methods, soil spectroscopy is more cost-efficient as measurements can be carried out faster and various soil properties can be inferred from a single spectral measurement. Accordingly, soil spectroscopy has the potential to greatly increase the scope of soil sampling efforts through increased coverage in both space and time.

The capability of laboratory soil spectroscopy in the visible to near-infrared (VIS-NIR) and mid-infrared (MIR) to determine a wide variety of chemical, physical and biological soil properties has been established by an extensive body of research over the years (see reviews, e.g., in [1,2,4,5]). Since estimations of soil parameters with spectral data are based on multivariate calibrations, a large number of statistical modelling approaches that relate the soil information in the spectra to reference values acquired with traditional laboratory methods were evaluated in parallel (see [6,7]). These include—in addition to partial least squares (PLS) regression as the most commonly used method [8]—multivariate adaptive regression splines, artificial neural networks, support vector machines, or regression trees and random forests [9,10,11,12].

In addition to laboratory spectroscopy, portable soil spectroscopy has received increasing attention in recent years in the context of proximal soil sensing, which refers to a range of methods that aim to provide a rapid on-site characterization of soils [13]. With the recent availability of portable MIR instruments, the potential for on-site analysis of soils has increased markedly as the MIR region (2.5–25 μm) is sensitive to the fundamental vibrations of various main soil constituents, in contrast to the VIS-NIR region (0.4–2.5 μm), which had been available for on-site spectroscopic measurements previously, but was limited to the detection of considerably weaker overtone and combination bands.

Compared to laboratory spectroscopy, on-site spectral analysis with portable instruments can further increase the benefits of soil spectroscopy by minimizing the number of soil samples that have to be collected and transported to the lab, as well as the requirement for extensive sample pre-treatment, which most commonly consists of drying and grinding [14,15]. In addition, the on-site analysis of spectral measurements allows us to devise more effective and representative spatial soil sampling strategies as MIR spectra essentially represent a fingerprint related to chemical or physical soil properties and may thus be used to gather information about the spatial distribution of soil properties in advance. Portable MIR spectroscopy therefore represents a promising approach to enhance traditional soil mapping efforts.

Operational use of portable MIR spectroscopy for soil mapping requires spectral readings of sufficient quality to derive quantitative predictions of spectrally active key soil components, ideally comparable to bench-top instruments. The accuracy of spectral prediction models is linked to both the overall sensitivity of the measured spectra towards the studied soil properties as well as the specific in situ soil conditions. Reeves [16], for example, scanned field moist and dry soil samples with a portable MIR spectrometer and found some predictions of soil properties to be poor, presumably due to of the effects of soil water, which obscured the spectral impact of other soil constituents on the measured spectra. Similar observations regarding the effects of soil moisture on the prediction accuracies obtained from MIR data for organic matter, iron content, cation exchange capacity or bulk density were also made by Ji et al. [15]. These studies, however, did not include direct comparisons with bench-top instruments, which makes it difficult to separate technical capabilities, i.e., the quality of measurements that can be achieved with portable spectrometer technology, from environmental effects.

Further restrictions of portable MIR spectroscopy may arise from the type of instrumentation. For the collection of in situ soil data, the DRIFT (Diffuse Reflectance Infrared Fourier Transform) recording technique is most commonly used. DRIFT is applicable to surfaces of solid and particulate materials with low transmissivity. Furthermore, the DRIFT method does not require extensive sample preparation (no pellets or dispersive matrices are needed, only crushing of crusts or of stable soil aggregates may be necessary). DRIFT spectra are recorded by illuminating the sample with an artificial light source; the IR radiation penetrates the sample and undergoes absorption, transmission and scattering before eventually leaving the sample as diffusely reflected light, which is collected by a set of mirrors and then focused onto the detectors [17]. Energy throughput, detector type and the employed measuring protocol (sampling interval, spectral resolution, integration time or number of co-added scans) will influence the quality of the collected spectra [18] and might cause differences between data measured by portable and classical lab spectrometers.

Against this background, the overall goal of this study was to test a recently introduced commercial portable MIR spectrometer (Agilent 4300 Handheld FTIR Spectrometer; Agilent Technologies, Santa Clara, CA, USA) for its general use and performance in soil spectroscopy in comparison to an established lab spectrometer. We collected MIR spectra for a set of 40 soil samples using the Agilent instrument equipped with a DRIFT accessory and compared them to readings performed for the same samples with a Bruker Tensor 27 bench-top spectrometer. Measurements with the Bruker instrument were carried out with both a DRIFT accessory and a nitrogen-purged Ulbricht sphere. This integrating sphere allowed measurements of a sample’s directional hemispherical reflectance (DHR), which can be used to determine emissivity spectra by applying Kirchhoff’s law (with emissivity = 1 − reflectance) and thus allows quantitative comparisons with measurements acquired in passive mode as carried out in thermal infrared remote sensing [19].

Our test comprised three series of measurements on dried and ground soil samples with the Agilent instrument. Spectra were evaluated by (i) comparing their shape to that of the Bruker spectra and by (ii) quantification of the inherent noise estimated by wavelet decomposition. We thus focused on the comparison of portable and bench-top FTIR instruments under standardized laboratory conditions (air-dried samples, ground to particle sizes <100 μm) to exclude environmental effects that may arise from on-site measurements.

In addition, we explored whether quantitative prediction models for various physicochemical soil properties would achieve similar accuracies for spectra collected with different instruments and/or sampling technologies (Bruker DHR, Bruker DRIFT, Agilent DRIFT). We focused on five soil properties that are relevant for agricultural practices and constitute key indicators of soil health and fertility: soil organic carbon (SOC), total nitrogen (N) (both closely related to soil organic matter), soil pH, clay and sand content. These soil properties can typically be estimated from VIS-NIR and MIR spectra since they are either spectrally active with well-defined absorption bands (SOC, clay minerals) or may correlate highly with other spectrally active soil constituents [2,20]. Since exploring the potential of different multivariate calibration methods was not in the scope of this study, we used PLS regression for multivariate calibrations, which is the de-facto standard tool in soil spectroscopy due to its ease-of-use through implementation in all spectroscopic software packages and a comparably straightforward and efficient calibration and parameter tuning approach. Calibration models were evaluated in a repeated 10-fold cross-validation approach to estimate both model accuracy and precision. Finally, we identified and compared key wavenumbers and spectral regions relevant for the prediction of soil components between instruments and sampling technologies with a Monte Carlo based variable selection approach.

## 2. Materials and Methods

### 2.1. Study Site, Sampling and Soil Chemical Data

**Figure 1:**
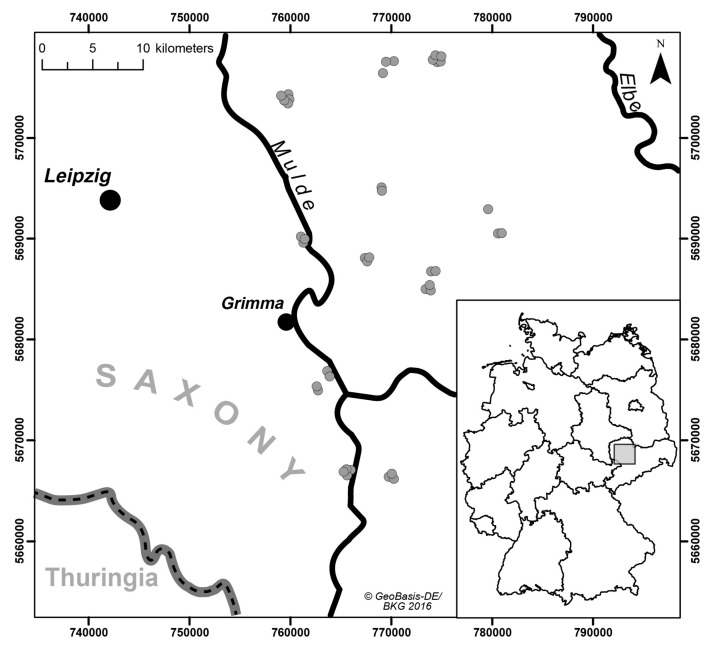
Overview of the study area in eastern Central Germany (State of Saxony) with soil sampling locations (coordinate system: UTM zone 32U, ETRS89).

We collected a set of soil samples with limited heterogeneity with respect to soil parent material. A limited heterogeneity is appropriate to avoid highly variable and usually complex spectral properties of soils with great differences in soil conditions [21], as this would have potentially masked systematic differences induced by the tested spectrometers and measurement settings. The sampling area was located in the German state of Saxony in the Northwest Saxon basin. We included a set of 40 agricultural plots where we took samples from the very top layer (0–5 cm depth in the Ap horizon) (Figure 1). Geologically, the sampled region is characterized by Permian bedrock geology (rhyolites and ignimbrites), Cretaceous-Tertiary weathering products (like kaolin) and quaternary sediments (loess, Pleistocene terrace gravel) [22]. Typically, the top soils in the selected study region are influenced by loess sediments with variable particle size following a gradient from sandy loess sediments in the north to more finely textured silty loess in the south. According to this spatial gradient, soil texture classes differed and ranged from loamy sand (*n* = 2), sandy loam (11), loam (5), silt loam (21) to silty clay loam (1) (classes according to FAO [23]).

For each soil sample, contents of SOC, N, clay and sand, and pH values were determined with standard laboratory methods. Prior to analysis, soil samples were air-dried, sieved (≤2 mm) and, depending on the analysis method, subsequently homogenized by careful grinding. The Köhn sieve-pipette method [24] was used to determine soil texture from the sieved soil material. We found contents of rock fragments or skeleton (>2 mm) to be low in all cases (less than 10 percent by volume). None of the soil samples contained carbonate-C. Total contents of SOC and N were measured by dry combustion of samples at 1100 °C and gas chromatography using a EuroEA elemental analyzer (HekaTech, Wegberg, Germany). Soil pH was measured potentiometrically in 0.01 M CaCl_2_ solution with a glass electrode.

Summary statistics for each soil parameter are given in Table 1. Values for SOC ranged from 0.62% to 2.70 with a standard deviation of 0.41%. Soil N was highly correlated with SOC (Pearson’s *r* = 0.90; significant at the *p* = 0.01-level) and also followed a similar distribution. High correlations (albeit markedly lower) were also found between clay and sand (*r* = −0.73) and between clay and N (*r* = 0.72) (both significant at the *p* = 0.01-level). Soil pH ranged from acidic (two samples with pH < 4.5) to slightly acidic to neutral (24 samples in the 6.0 ≤ pH ≤ 7.3 range) conditions. The large spread in contents for sand (3.5–82.4%) and clay (6.8–35.9%) reflects the large diversity in soil texture ranging from loamy sand to silty clay loam. Overall, the collected sample set provided a usable, albeit limited range for SOC and N for multivariate calibrations, while the limited number of covered soil types and parent materials ensured that calibration models would be more likely driven by individual absorption bands instead of primarily by soil type dependent spectral profiles.

### 2.2. Spectral Data

Spectral data in the MIR region were acquired with bench-top (Bruker Tensor 27; Bruker Optik GmbH, Ettlingen, Germany) and handheld (Agilent 4300 Handheld FTIR) instruments on sieved (<2 mm), dried (lab oven at 40 °C) and then ball-milled soil samples (finely ground for 5 min to achieve particle sizes <100 μm and to homogenize the sample). A detailed overview of the different measurement configurations for each instrument is provided in Table 2.

Five series of measurements were collected in total. On the Bruker instrument (equipped with a deuterated, L-alanine doped triglycine sulfate (DLaTGS) detector and an extended range KBr beam-splitter), we measured DHR spectra using a nitrogen purged integrating sphere and DRIFT spectra with a diffuse reflectance accessory (EasyDiff, Pike Technologies, Madison, WI, USA). With both accessories, we performed 200 scans of the sample and 200 scans of the background with a spectral resolution of 4 cm^−1^, corresponding to a 2 cm^−1^ sampling interval. The spot diameter was about 20 mm for DHR and 6 mm for DRIFT measurements.

For the handheld instrument, equipped with a deuterated triglycine sulfate DTGS detector and a zinc selenide beam splitter, we collected three series of measurements with the instrument’s diffuse reflectance interface (spot diameter about 2 mm only). These series were collected by three different operators (Agilent #1, #2, #3) as we expected its performance to be more variable in comparison to the standardized bench-top instrument measurements. Each series of measurements for the Bruker and Agilent instruments was generated by pooling individual spectra collected on two subsamples taken from the bulk soil sample.

**Figure 2:**
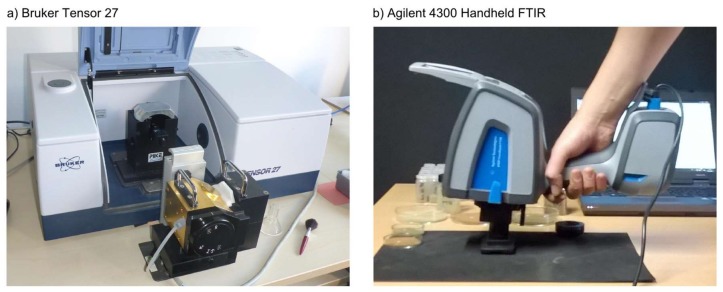
MIR spectrometers used in this study: (**a**) Bruker Tensor 27 bench-top instrument with EasyDiff diffuse reflectance accessory (back, in the sample compartment) and Ulbricht sphere (front); (**b**) Agilent 4300 Handheld FTIR measurement with custom sample cup.

Diffuse reflectance measurements with the handheld spectrometer were carried out with a custom sample setup (Figure 2). Soil samples were filled into a small sample cup (~1 cm diameter) and put into a custom sample holder, which provided some guidance to stabilize the instrument and ensured a consistent measurement angle and distance between soil sample and sampling interface while holding the instrument (~0° zenith angle, distance < 0.5 mm). We opted for a simple sampling setup and manual operation of the handheld instrument, in contrast to a larger fixed stage, since portability and potential on-site use in the field are the main advantages of handheld instruments and the sampling approach suggested here is similar to what might be achieved under field conditions. Integration time (number of co-added scans) was adjusted following the same rationale. For each spectral measurement, 64 individual scans were averaged as a compromise between signal-to-noise ratio and scan duration (~1 min for 64 co-added scans). To test the potential benefits of additional scans, however, we also pooled all three 2 × 64 measurements into a single composite spectrum (Agilent #4) with a total number of scans comparable to the bench-top instrument measurements.

Handheld MIR data were collected in the 4000–650 cm^−1^ frequency range with 4 cm^−1^ spectral resolution (sampling interval ~2 cm^−1^); prior to each measurement, a background spectrum was taken with a gold-plated reference cap, which is part of the diffuse reflectance interface provided by the manufacturer. The background spectrum was collected to compensate for the effects of potential instrument drift and variation in the lab environment, respectively (e.g., changes in the composition of the local atmosphere). To remove these effects, the measured signal was divided internally by the collected background spectrum.

For further statistical analysis, we used the spectra in the domain between 4000 and 650 cm^−1^, as this spectral range was available for all instruments and settings. Spectra were resampled to a regular increment of 2 cm^−1^ using the shape-preserving Piecewise Cubic Hermite Interpolating Polynomial approach implemented in MATLAB (see, e.g., Moler [25]). Pre-processed spectra therefore consisted of 1676 data points *x*_i_ (with *i* = 650, 652, …, 4000 cm^−1^), measured reflectances (R) were transformed to (pseudo-)absorbances by −log_10_(R).

### 2.3. Wavelet Analysis

Mid-infrared spectra collected with handheld instruments may be expected to contain higher noise levels, i.e., unsystematic high frequency variation across the signal, than those collected with bench-top instruments due to smaller spot size, manual handling of the instrument and lower energy throughput. To estimate the noise fraction in individual spectra, we employed wavelet analysis to extract the high frequency components from the complete signal.

With the application of wavelets, spectra or signals can be decomposed into sets of coefficients approximating shifted and scaled versions of a mother wavelet. In continuous wavelet transforms (CWT), the analyzing wavelet is shifted over the full domain of the signal. At each position, coefficients are calculated which represent the correlation between wavelet and the respective section of the signal. CWT therefore provides many wavelet coefficients as a function of position and scale (*a*). As scale (wavelet width) increases, the finer details of the signal get lost. In consequence, the CWT coefficients at the smallest scale (*a* = 1) are a good estimate of the noise level in the spectra [26,27].

For the analysis of measured spectra, a great number of different mother wavelets may be considered. Absorption features in the spectra are often similar to the Gaussian function or a combination of multiple Gaussian functions, so we applied the Mexican hat wavelet, which is proportional to the second derivative of the Gaussian probability density function. As mentioned above, we used the decomposition at *a* = 1 to quantify noise contained in the spectra of the different measurement series.

### 2.4. Partial Least Squares Modeling

The predictive power of the different measurement series was evaluated by calibrating multivariate regression models for SOC, N, clay content, sand content and pH values based on components (latent variables) that were extracted from the measured spectra by the non-linear iterative PLS algorithm. Values of SOC, N and clay were transformed beforehand by log_10_, pH values were squared, to correct for skewness in the untransformed distributions. Transformed values could be well approximated with normal distributions (Shapiro-Wilk test, *p* > 0.20). Final estimates were re-transformed to the original data space. Values for sand content were not transformed, as the statistical distribution of sand content was bimodal and a transformation was thus inappropriate.

To assess both the accuracy and the precision of calibrations with PLS regression, we carried out 1000 runs of repeated 10-fold cross-validation (CV) for each measured series and each soil parameter; groups of samples used for CV were randomly defined. The optimal number of latent variables (*n*_opt_ l.V.) was chosen for each model by means of the root mean squared error of cross-validation (*RMSE*) and the Akaike Information Criterion (AIC) [28], calculated according to Viscarra Rossel and Behrens [6]:

*AIC* = *n* ln(*RMSE*) + 2*p*, (1)

with *n* as the number of soil samples used for calibration (*n* = 40 in our case) and *p* as the number of latent variables used in the respective model. The maximum possible number of latent variables was set to *p* = 8, the minimum AIC score then indicated the best model as a trade-off between accuracy and model parsimony. All calculations were done using the freely available libPLS software coded in MATLAB [29], which we modified for our specific purposes.

We used different metrics to judge accuracies obtained in the CV approaches; *RMSE*, the coefficient of determination (*r*^2^) and the ratio of performance to deviation (*RPD*); *RPD* is defined as the ratio of the standard deviation of chemically measured reference values to the *RMSE* obtained in the CV.

### 2.5. Identification of Spectral Key Variables

For the identification of spectral key variables—performed for each soil property and for each measurement series of the Bruker and Agilent instruments—we used the competitive adaptive reweighted sampling (CARS) method, which is described in detail by Li et al. [30].

CARS, combined with a regression approach such as PLS regression, aims at selecting key variables in a computationally efficient procedure. A series of *j* sampling runs is performed, each including two selection steps. An exponential function (based on *j* and the number of original spectral variables as parameters) is used to define the number of spectral variables to be kept in the first step, i.e., the spectral variables are sorted according to their regression coefficients and all variables beyond the defined maximum number are eliminated. In the second step, *n* random sampling runs are carried out to select another subset from those *n* variables that were kept beforehand, whereby the selection probability of each variable again depends on its regression coefficient. In this step, only those variables that were picked more than once (sampling with replacement) are retained. At the end of all sampling runs, *j* models have been defined. From these models, the best is chosen using the minimum *RMSE* in the CV and the spectral variables used in this final model are considered to be key variables.

For a robust identification of key variables, we varied the data subjected to CARS and performed 1000 repetitions. In each repetition, only a subset of the complete dataset was selected by random sampling (covering 36 samples or 90% of the original sample set) and used in the procedure described above. In all repetitions and sampling runs, the maximum number of latent variables used in the regression approach was fixed to *n*_opt_ l.V. found in the 10-fold CV approach carried out with full spectrum PLS regression beforehand (as described in Section 2.4). At the end of all repetitions, we analyzed how often each variable had been included in the total of all 1000 “best” models. Variables selected with high frequency can be considered to be more robust to sample variation or noise [30], so that we used these cumulated frequencies to compare selection patterns between the different measurement series. For the described Monte Carlo CV CARS approach, based on a subset of training samples with a large number of repetitions, we used the MATLAB function included in the libPLS software package (“carspls_mccv”).

## 3. Results

### 3.1. Spectral Data Quality

**Figure 3:**
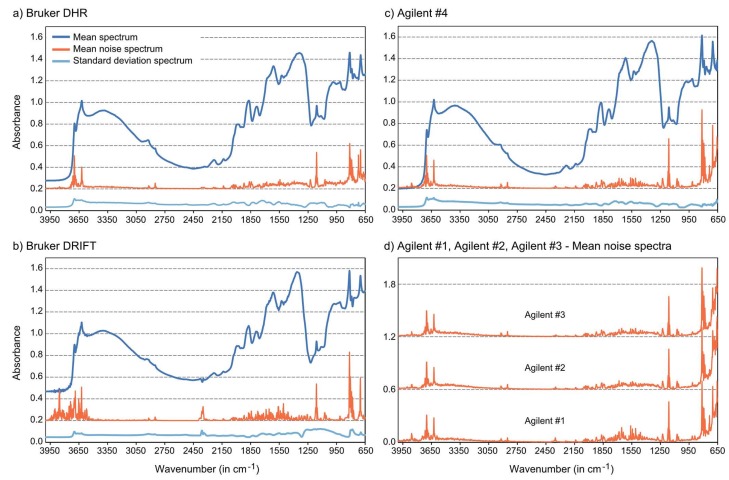
Characteristics of the measured spectra. (**a**–**c**) Mean, noise and standard deviation spectra for Bruker (**a**,**b**) and composite Agilent (#4) (**c**) data (noise values multiplied by a factor of ten and with an offset of 0.2 for clarity), (**d**) Noise spectra of all three individual Agilent measurement series (multiplied by a factor of ten and with an offset of 0.6 (Agilent #2) and 1.2 (Agilent #3), respectively).

For all measurement series, we calculated mean, standard deviation and noise spectra, the latter extracted from the wavelet analysis (Figure 3). For the Agilent instrument, the basic shape of the mean spectra was very similar between the different measurement series, so only the mean and standard deviation spectrum of the composite series Agilent #4 are illustrated, but the noise spectra of all datasets are shown.

The recorded MIR spectra exhibited varying ranges of absorbance values. For Bruker, DRIFT spectra ranged from 0.45 to 1.58 and DHR spectra from 0.28 to 1.46; Agilent spectra showed the greatest minimum to maximum differences (Agilent #4: 0.20 to 1.62). Positions of maxima (Bruker DRIFT and Agilent #4 814 cm^−1^, Bruker DHR 812 cm^−1^) and minima (3852 cm^−1^ for Bruker DRIFT, otherwise 4000 cm^−1^) coincided well. The shape of the measured spectra showed a good match especially between Bruker DHR and the pooled Agilent (#4) series. In the case of the Bruker DRIFT spectra, some features were different, in particular the shape of the feature between 1070 and 1280 cm^−1^ (i.e., the generally “w”-shaped silicate inversion band [31]), the feature peaked at around 1350 cm^−1^ (being less broad in the Bruker DRIFT than in the DHR and Agilent spectra) and a small noisy feature at 2350 cm^−1^ (most likely due to atmospheric CO_2_ [32]). Moreover, the feature at 2920 cm^−1^ was more pronounced in the Bruker DHR and Agilent spectra than in the Bruker DRIFT spectra (Figure 3a–c).

As an overall measure of spectral quality, we cumulated the extracted noise (in absorbance) for all 40 spectra and over all wavenumbers (652–4000 cm^−1^), which provided a ranking of Bruker DRIFT spectra (cumulated noise = 156) < DHR (206) < Agilent #4 (211) < Agilent #1 (273) < Agilent #3 (275) < Agilent #2 (304). Noisy regions differed between instrumentation and settings. Bruker DRIFT spectra, for example, showed markedly more noise than DHR or Agilent spectra in the regions greater than 3550 cm^−1^, but generally less noise (with the exception of some pronounced peaks) in the region between 652 and 1400 cm^−1^ (Figure 3b). Agilent spectra were definitely noisier than Bruker DRIFT and DHR spectra in the 652–850 cm^−1^ region (Figure 3c), so that the ranking concerning noise without this spectral region (850–4000 cm^−1^) switched to Bruker DRIFT (cumulated noise = 121) < Agilent #4 (135) < DHR (153) < Agilent #3 (175) < Agilent #1 (180) < Agilent #2 (190). The Agilent series (Agilent #1–#3) revealed slight differences concerning noise level, but the general noise pattern kept very stable (Figure 3c,d). The averaging of measured Agilent spectra to the composite series Agilent #4 was beneficial in terms of a markedly reduced noise level.

### 3.2. PLS Calibration Results

Estimates of all soil variables reached, with the only exception of soil pH, *r*^2^ values ≥ 0.85 and *RPD* values > 2.50, each with the best spectral dataset provided in all four cases by the Bruker instrument with integrated sphere measurements (Table 3). One of the Agilent series (Agilent #2) provided the highest accuracies for pH, which were slightly inferior to those of the other variables with *r*^2^ at 0.78 and *RPD* at 2.15. Predictive advantages of DHR spectra regarding the other datasets were most pronounced for SOC (in terms of *r*^2^—0.85 compared to 0.80 for Agilent #3) and for sand content (in terms of *RPD*—3.99 compared to 2.95 for Agilent #4).

For the Agilent measurements, the highest accuracies were obtained—depending on the soil property—with different datasets. Accuracies were highest with Agilent #2 for N and pH, with Agilent #3 for SOC and with Agilent #4 for clay and sand contents. In general, differences of accuracies achieved with Agilent data ranked first and second were small in all cases (Table 3). Results achieved with Bruker DRIFT spectra were poorest for SOC and N. For the other variables DRIFT spectra ranked fourth (clay, sand) and fifth (pH). Compared to DHR spectra, *RMSE* increased in all cases by more than 20%.

**Figure 4:**
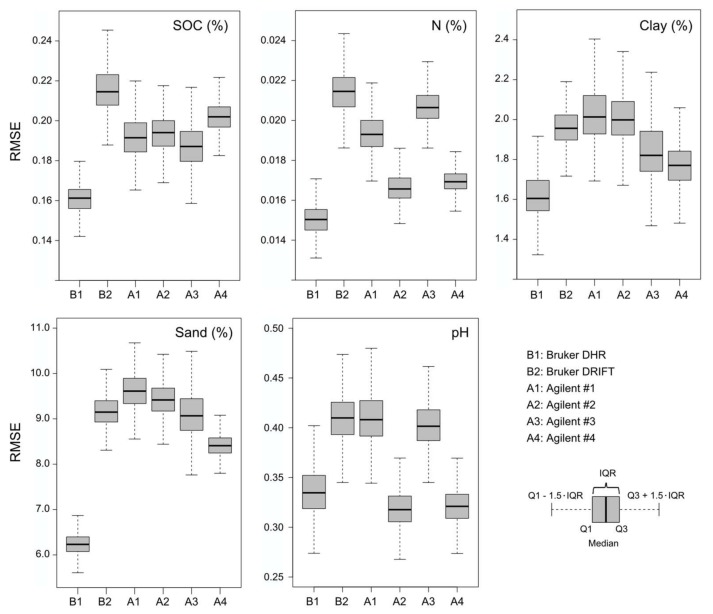
Distributions of *RMSE* values across repeated 10-fold CV PLS runs for SOC, N, clay content, sand content and pH values for all measured series.

Distributions of *RMSE* values for each soil property from the 1000 CV runs also indicate that DHR measurements yield the most robust estimations of SOC, N, clay and sand contents (Figure 4). For these soil parameters, the interquartile ranges (IQR) of the *RMSE* values for the DHR series do not overlap with the IQRs of the other measurement series, i.e., the third quartile of the DHR *RMSE* values is lower than even the first quartile of *RMSE* values in all other cases. Ranking for the Agilent series differed across soil properties; Agilent #2 and Agilent #4, however, each performed best for two soil properties. The averaging of measured Agilent spectra to Agilent #4 provided more robust estimates indicated by generally smaller interquartile *RMSE* ranges compared to Agilent #1–#3. The use of Agilent #4 data was markedly more successful than using Bruker DRIFT spectra, as the upper quartile of Agilent #4 *RMSE* values was lower than the lower quartile of Bruker DRIFT *RMSE* data in all cases (Figure 4).

In summary, Bruker DHR measurements tended to yield the best calibration models both in terms of predictive accuracy and precision. The Agilent #4 series showed similar robustness, whereas Bruker DRIFT and the individual Agilent series #1–#3 provided much more variable calibration results. While DRIFT measurements with the Agilent handheld instrument tended to allow better calibrations than Bruker DRIFT, there was overall a larger difference in calibration results between DHR and DRIFT measurements than across the DRIFT datasets collected with the bench-top and handheld instruments.

### 3.3. Key Wavenumbers and Relevant Spectral Regions

**Figure 5:**
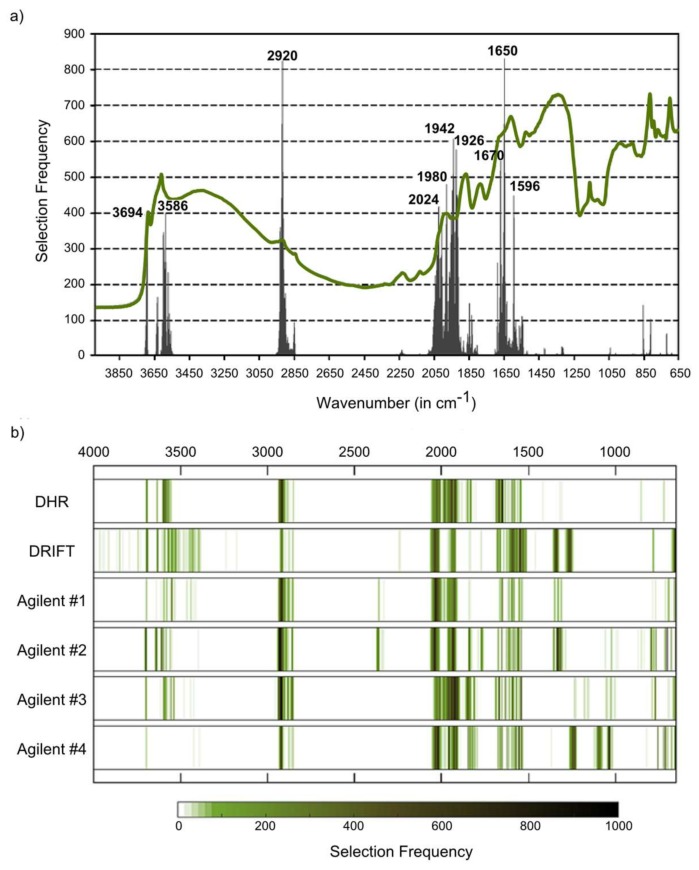
Key wavenumbers for SOC calibrations: (**a**) selection frequencies found for wavenumbers of the DHR measurement series (peaks labelled) overlaid with mean DHR spectrum; (**b**) heat map of SOC selection frequencies for all measured series.

Based on the applied Monte Carlo CV approach with the CARS spectral variable selection algorithm, we identified key wavenumbers and key spectral regions for each soil property and each measurement series. For SOC and DHR measurements, we found key wavenumbers in the X−H stretching region from 4000 to 2500 cm^−1^ and in the triple- and double-bond regions from 2500 to 1500 cm^−1^, but not in the fingerprint region (<1500 cm^−1^) (Figure 5, Table 4).

Most prominent selection peaks were found around 1650 cm^−1^, 2920 cm^−1^ and between 1918 and 1942 cm^−1^. These peaks did not match with those peaks that we found with Bruker DRIFT spectra. There, a total of five regions at wavenumbers less than 1600 cm^−1^ were of high importance, and only two regions at higher wavenumbers (2022–2048 cm^−1^, 3692–3696 cm^−1^) coincided at least in parts with DHR key regions (Figure 5, Table 4).

Agilent spectral data showed some differences in the obtained selection patterns across the four series. Most markedly, we found three selection peaks in the fingerprint region for Agilent #3 data, located around 1036, 1092 and 1240 cm^−1^ and all without a match in the other Agilent series. Agilent #2 data did not provide any peak in this region, while selection frequencies from Agilent #3 data were markedly low in the X−H stretching region between 3000 and 4000 cm^−1^. All Agilent series provided prominent peaks in the 1920–1930 cm^−1^ and in the 2918–2922 cm^−1^ region, Agilent #1, #3 and #4 matched with a peak at 2018–2038 cm^−1^. Agilent #4 data, obtained by pooling Agilent #1, #2 and #3, provided the most parsimonious models with a mean number of only 35 selected spectral variables per run. In comparison, the mean number of used spectral variables was 60, 57, 50 for Agilent #1–#3, 56 for Bruker DRIFT and 45 for DHR spectra (Figure 5). Heatmaps of selection frequencies (Figure 5) and listed key regions (Table 4) indicate a closer match between Agilent #4 and Bruker DHR spectra than between Bruker DRIFT and Bruker DHR spectra.

**Figure 6:**
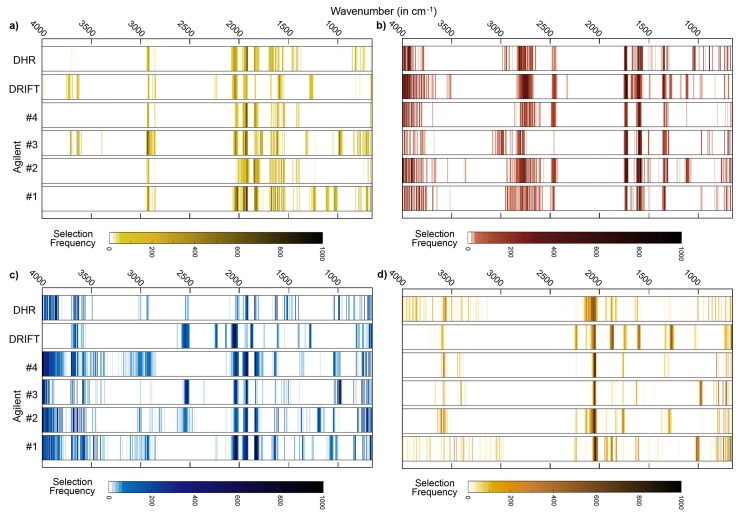
Heatmaps of wavenumber selection frequencies for (**a**) N, (**b**) pH, (**c**) clay content and (**d**) sand content provided by the applied Monte Carlo CV approach with the CARS spectral variable selection algorithm.

In line with the results obtained for SOC, the Agilent #4 series provided the most parsimonious models also for N, pH and sand (but not for clay). Generally, the modeling of N and sand contents made use of a low number of spectral variables compared to SOC, pH and clay contents (Figure 5 and Figure 6). Selection patterns demonstrated similarities between SOC and N. Absorbances at 2920 cm^−1^ and at 1926–1928 cm^−1^ were identified as key spectral regions in DHR and Agilent spectra for the estimation of both soil variables (Figure 5 and Figure 6a, Table 4); absorbances at 1650–1652 cm^−1^ were found to be relevant for SOC and N with DHR, at 2032–2040 cm^−1^ with Bruker DRIFT spectra (Table 4).

Minor overlaps could also be found for SOC, clay and sand. For example, absorbances at 2044–2048 cm^−1^ were relevant for all three constituents in the case of Bruker DRIFT spectra. Further overlaps between key regions for clay and sand contents, however, were not evident. Also, for Bruker DRIFT spectra, the spectral regions at 652–660 cm^−1^ and at 1264–1272 cm^−1^ were considered to be relevant for SOC and sand (Table 4).

The spectral region between 1926–1932 cm^−1^ was highly important for SOC and clay when considering Agilent #4 spectra (Table 4). Among the considered soil variables, pH values were outstanding concerning their spectral selection pattern. Spectral key regions did not show overlaps with key regions of other soil variables. In all measurement series, spectral variables in the region between 800 and 1400 cm^−1^ were irrelevant for estimating pH values and thus neglected in the selection procedure (Figure 6b), while this region showed prominent peaks for all other soil variables as described above and also indicated by Figure 5 and Figure 6a,c,d.

## 4. Discussion

Although we found different noise levels from one instrumental configuration to the other (and between the different measurement series with the Agilent instrument) we could not identify a direct relationship between spectra quality and obtained estimation accuracies. For example, the Agilent #2 series with a relatively high noise level succeeded over Agilent #1 and #3 data for estimating N and pH values; Bruker DRIFT spectra, characterized by a low noise level between 650 and 3550 cm^−1^, provided the poorest estimates for SOC and N. This is in line with the findings of Soriano-Disla et al. [18], who compared different MIR instruments (reference laboratory and handheld instruments) and also stated that the quality of the spectra did not play a major role for the prediction of soil properties. Beyond noise, we found some minor differences in the shape of the spectra, especially between DRIFT spectra on the one hand and Bruker DHR and Agilent spectra on the other hand. This seemed to be more relevant than noise, especially in the case of sensitive regions; for example, the feature at 2920 cm^−1^, which is in the case of missing carbonates diagnostic to aliphatic CH and thus directly related to organic matter in soils [33], was less pronounced in the Bruker DRIFT spectra. Accordingly, this region was less frequently selected with these DRIFT spectra than with DHR or Agilent DRIFT spectra for the modeling of SOC and N.

For the studied soil properties, further typical features exist in the MIR region that may be used for qualitative or quantitative approaches, but only some of these diagnostic features can be identified in soils without incineration, chemical extraction or the application of fractionation techniques [31]. The clay fraction is most markedly linked to the region between 3600 and 3700 cm^−1^ (usually with a sharp peak at 3620 cm^−1^ caused by hydroxyl stretching [34]). According to this, key wavenumbers for clay contents were found for all datasets in this spectral region. Other known clay mineral features such as those centered between 1000 and 1100 cm^−1^ (Si−O stretching frequencies) [35] were not evident as key regions, independent from the considered instrumental setup. Quartz and feldspars may be used as spectrally active constituents to quantify the sand fraction [36]. Quartz can be detected by the “w”-shaped silicate band and by a typical doublet at 800 and 780 cm^−1^ [37,38], feldspar features are located at 600, 750 or 950 cm^−1^ [9]. All these features located at wavenumbers less than 1300 cm^−1^ did not correspond to one of the identified key regions (neither for clay nor for sand) although, for example, the “w”-shaped silicate band was markedly pronounced in all measured spectra. The region below 1000 cm^−1^, however, is a mixture of organic and mineral bands and thus may be better resolved by a subtraction of the ash soil spectrum from the untreated soil spectrum [39].

Important organic matter features are located at 2920 cm^−1^ (as described above), at 1740–1698 cm^−1^ (C=O groups in carboxylic acids, aldehydes and ketones), at 1640–1600 cm^−1^ (amide I band) and at 1575–1400 cm^−1^ (amide II band) (see compilation in Vohland et al. [40]). The region between 1740 and 1600 cm^−1^, which can be used to indicate hydrophilic organic components [41], was identified as a key region for SOC (with Bruker DHR spectra) and N (Bruker DHR and Agilent spectra). The amide II band was found to be relevant for SOC when using Bruker DRIFT spectra. For all instrumental configurations, absorbances at 2024–2026 cm^−1^ were identified as being relevant for the quantification of SOC, but a clear physical interpretation is missing. This region is, possibly linked to the silicate region between 1790 and 2000 cm^−1^, typically with three characteristic peaks (Figure 3a–c). Absorbances in this region are often (negatively) correlated with SOC and N [31], and we found this region to be a key spectral region for both constituents (on Bruker DHR and Agilent spectra, on Bruker DRIFT spectra only for N).

Similarity of the spectra and a good agreement of physically evident spectral key regions indicate a similar applicability of Bruker DHR and Agilent spectra for the quantification of both SOC and N and suggest a different performance of Bruker DRIFT spectra. This corresponds to the results obtained in the 10-fold CV approach, with decreasing accuracies for both soil variables ranked Bruker DHR > Agilent > Bruker DRIFT spectra (Table 3).

Results of the calibration approach crucially depend on the representativeness of both the chemically analyzed and the spectrally measured subsamples. Especially when modeling soil variables with a narrow concentration range, errors of the chemical reference method may affect estimation results markedly [18]. For the representativeness of the spectral measurements, Agilent DRIFT measurements were affected by the small spot diameter (2 mm) compared to Bruker DRIFT (6 mm) and DHR (20 mm) measurements. It is thus highly recommendable to repeat measurements with the Agilent device at different subsamples and to pool these data to one dataset, as done in the case of the Agilent #4 data. Results indicate that this improved spectra quality with an overall reduced noise level, resulted in more robust estimation models (lower variability of obtained *RMSE* values) and induced parsimony with respect to the selected spectral variables with a more distinct discrimination of important and unimportant ones.

As far as we know, this study is the first to test the Agilent 4300 Handheld FTIR in a direct comparison with a bench-top spectrometer. Soriano-Disla et al. [18], however, tested two other predecessor Agilent handheld FTIR devices (Agilent 4100 ExoScan, Agilent 4200 FlexScan), also operated with a DRIFT accessory, and compared them to a Frontier spectrometer (Perkin Elmer, Waltham, MA, USA) also combined with a DRIFT accessory as a bench-top reference instrument. In the calibration of SOC, pH, N, sand and clay they found only small differences between the different instruments with a maximum for N obtained with the portable FlexScan instrument (*r*^2^ = 0.75, *RPD* = 1.98) in comparison to the reference Frontier spectrometer (*r*^2^ = 0.70, *RPD* = 1.83). Across all studied properties (*n* = 17) the FlexScan performed best (with again small differences). Based on these results it was concluded that a portable MIR instrumentation can provide similar performance to bench-top instruments so that there is a high potential for in-field applications. Our study is in line with these results and supports this conclusion for the Agilent 4300 instrument, although obtained accuracies were inferior to the “best case” configuration realized with Bruker DHR measurements (highly sensitive DLaTGS detector with a high signal-to-noise ratio (minimum value > 6000:1), integrating sphere to measure hemispherical reflectances, 2 × 200 scans per sample, 20 mm spot diameter). In the direct comparison using a DRIFT accessory, at least the composite data of the Agilent 4300 instrument (Agilent #4 with 3 × 2 × 64 scans per sample) provided generally higher estimation accuracies for the studied soil properties than the Bruker DRIFT series, despite slightly noisier spectra.

## 5. Conclusions

Our findings show that handheld MIR spectrometers, in particular the Agilent 4300 Handheld FTIR tested in this study, can record soil spectra of comparable quality to bench-top instruments. Multivariate calibrations for various soil properties based on bi-directional DRIFT measurements with the handheld instrument were as good as or slightly better than the results achieved with DRIFT measurements from the Bruker instrument. Directional hemispherical reflectance measurements with an integrating sphere, however, yielded the best results and thus may be considered a reference method for stand-alone lab applications in soil spectroscopy. Since our study covered a comparably homogeneous set of agricultural soil samples from a single soil region, future research efforts to cover a broader range of soil types derived from more diverse parent materials and under different land use practices will be important to further generalize our findings.

The wavelet analysis of the very high frequency components of the MIR spectra suggests that the contamination of spectral data with minor measurement noise has little influence on the accuracy of multivariate calibrations. Accordingly, an increase in the integration time for individual measurements beyond the tested 64 co-added scans may be of limited practical use for on-site applications. In comparison, repeated measurements on different soil sub-samples appear more promising to derive representative spectra and stable calibrations.

We are currently in the process of investigating the performance of the Agilent 4300 Handheld FTIR instruments for on-site spectral data acquisition. For on-site applications, it will be important to consider that MIR spectroscopy is also impacted by additional variability in soil moisture and more pronounced soil sample heterogeneity compared to dried and crushed samples analyzed in the laboratory.

## Figures and Tables

**Table 1:** Summary of chemical data for the studied soil samples (*n* = 40).

	Min ^1^	Q1 ^1^	Median	Q3 ^1^	Max ^1^	Mean	sd ^1^	Skewness
pH	4.15	5.55	6.14	6.49	7.17	5.98	0.685	−0.84
SOC (%)	0.62	1.12	1.35	1.64	2.70	1.39	0.41	0.83
N (%)	0.048	0.105	0.125	0.152	0.291	0.133	0.046	0.99
Clay (%)	6.8	11.0	14.4	17.9	35.9	15.0	5.6	1.45
Sand (%)	3.5	14.3	31.2	62.6	82.4	38.1	24.9	0.43

^1^ Metrics: minimum (min), first quartile (Q1), third quartile (Q3), maximum (max) and standard deviation (sd).

**Table 2:** Overview of collected MIR data and instrument configurations.

Measurement Series	Instrument	Interface	Co-Added Scans	Frequency Range (in cm^−1^)	Background
Bruker DHR	Bruker Tensor 27	Ulbricht sphere	2 × 200	7000–370 ^2a^	Gold reference background
Bruker DRIFT	Bruker Tensor 27	DRIFT	2 × 200	7000–370 ^2a^	Blank sample compartment
Agilent #1	Agilent 4300	DRIFT	2 × 64	4000–650 ^2b^	Gold-plated reference cap
Agilent #2	Agilent 4300	DRIFT	2 × 64	4000–650 ^2b^	
Agilent #3	Agilent 4300	DRIFT	2 × 64	4000–650 ^2b^	
Agilent #4	Agilent 4300	DRIFT	3 × 2 × 64 ^1^	4000–650 ^2b^	

^1^ Pooled from Agilent #1, Agilent #2 and Agilent #3; ^2a^ corresponds to a wavelength range from 1.429 to 27.027 μm; ^2b^ corresponds to 2.5 to 15.385 μm.

**Table 3:** PLS regression results (averaged from 1000 runs of 10-fold CV with best results for each soil property in bold).

	DHR	DRIFT	Agilent #1	Agilent #2	Agilent #3	Agilent #4
**SOC (%)**						
*number l.V.* ^a^	**5**	7	7	5	6	5
*r*^2^	**0.85**	0.73	0.78	0.78	0.80	0.77
*RMSE*	**0.16**	0.22	0.19	0.19	0.19	0.20
*RPD*	**2.58**	1.92	2.16	2.14	2.21	2.05
**N (%)**						
*number l.V.* ^a^	**5**	7	7	4	6	4
*r*^2^	**0.89**	0.79	0.82	0.87	0.80	0.87
*RMSE*	**0.015**	0.022	0.019	0.017	0.021	0.017
*RPD*	**3.08**	2.16	2.39	2.78	2.24	2.72
**Clay (%)**						
*number l.V.* ^a^	**7**	8	8	8	5	5
*r*^2^	**0.92**	0.88	0.87	0.87	0.89	0.90
*RMSE*	**1.61**	1.96	2.03	2.02	1.86	1.77
*RPD*	**3.48**	2.86	2.77	2.79	3.03	3.17
**Sand (%)**						
*number l.V.* ^a^	**5**	7	5	4	7	4
*r*^2^	**0.89**	0.79	0.82	0.87	0.80	0.87
*RMSE*	**6.25**	9.19	9.65	9.44	9.13	8.43
*RPD*	**3.99**	2.71	2.58	2.64	2.74	2.95
**pH**						
*number l.V.* ^a^	8	8	7	**7**	7	6
*r*^2^	0.76	0.67	0.65	**0.78**	0.67	0.78
*RMSE*	0.34	0.41	0.41	**0.32**	0.40	0.32
*RPD*	2.03	1.68	1.67	**2.15**	1.71	2.14

^a^ Number of latent variables.

**Table 4:** Spectral key regions identified from Monte Carlo CV CARS runs.

		4000–2500 cm^−1^ ^a^	2498–1500 cm^−1^ ^b^	<1500 cm^−1^ ^c^
SOC	DHR	2916–2924, 3586, 3692–3694	1594–1596, 1648–1652, 1670–1672, 1918–1948, 1978–1980, 2024–2026	−
	DRIFT	3630, 3692–3696	1544–1554, 1582–1598, 2022–2048	652–662, 1256–1272, 1332–1348
	Agilent #4	2912–2926	1926–1932, 1940, 2018–2044	−
N	DHR	2916–2918	1406, 1650–1652, 1676–1678, 1844–1848, 1916–1928, 1938–1942, 2042–2044	−
	DRIFT	−	1588–1596, 1928, 2032–2050	−
	Agilent #4	2918–2922	1538–1540, 1612–1614, 1684, 1834–1846, 1916–1940	−
pH	DHR	2806, 2814, 3966–3974	1608–1614, 1730–1742	670, 704, 774, 888, 944–946, 1044, 1354–1356
	DRIFT	2740–2744, 2760–2762, 3856, 3904–3906, 3932–3952, 3970–3978	1598–1602, 1722–1746	1350–1352
	Agilent #4	2736, 2756, 2782, 3824–3826, 3860, 3890, 3906–3908, 3934, 3954–3956, 3976, 3982	1592–1610, 1730–1736	1344–1346
clay	DHR	3676, 3852–3868, 3926–3928	1632–1634, 1842–1850	658, 706, 734, 986–988, 1402
	DRIFT	3644, 3666–3668	1874–1876, 2032–2064	680–682
	Agilent #4	3678, 3698–3700, 3934, 3946, 3956, 3964, 3984–4000	1834–1840, 1924–1934	712
sand	DHR	3556–3560	2042–2082	670, 1000
	DRIFT	−	1598–1600, 1862–1870, 2044–2058	652–660, 1264–1276
	Agilent #4	3568–3570, 3584–3588	1760, 2036–2066	660, 714, 810–816

^a^ X–H stretching region; ^b^ triple- and double-bond regions; ^c^ fingerprint region.
